# Using Citric-Acid-Based Anodization to Form Magnesium-Doped Carbonated Apatite-Containing Oxides on Solid and 3D-Printed Titanium Substrates

**DOI:** 10.3390/jfb17040190

**Published:** 2026-04-14

**Authors:** Amisha Parekh, Arunendu Ettuthaiyil Sambasivan, Mikyle Paul, Arash Soltani, Aya Ali, John Tucker, Jonathan W. Pegues, Nima Shamsaei, Amol V. Janorkar, Michael D. Roach

**Affiliations:** 1 Department of Biomedical Materials Science, University of Mississippi Medical Center, Jackson, MS 39216, USA; amishaparekh96@gmail.com (A.P.); asambasivan@umc.edu (A.E.S.); ayagneady.1@gmail.com (A.A.); jtucker8989@yahoo.com (J.T.); ajanorkar@umc.edu (A.V.J.); 2 Department of Mechanical Engineering, Auburn University, Auburn, AL 36849, USA; mzp0114@auburn.edu (M.P.); asoltani@astm.org (A.S.); jonathan.pegues@amaeroinc.com (J.W.P.); shamsaei@auburn.edu (N.S.); 3 National Centre for Additive Manufacturing Excellence (NCAME), Auburn University, Auburn, AL 36849, USA

**Keywords:** 3D-printed, titanium, hydroxyapatite, anodization, implants, bone, stress shielding, surface modification, bioactivity, ion release

## Abstract

With increasing life expectancy and an aging global population, the demand for orthopedic and dental implants is increasing. Recently developed, citric-acid-based anodization processes facilitate the production of more bioactive oxide layers by incorporating important bone minerals such as Ca, P, and Mg and forming bone-like crystalline compounds such as carbonated apatite on titanium implant materials. The primary goal of the present study was to evaluate the applicability of these anodization processes to solid and 3D-printed titanium alloy substrates. The anodized oxides produced on each solid or 3D-printed lattice substrate revealed multi-scaled surface roughness profiles as evidenced by scanning electron microscopy, optical microscopy, and surface roughness analyses. Additionally, each oxide group was shown to incorporate substantial amounts of Ca, P, and Mg bone-mineral dopants and form AB-type carbonated apatite, as shown using a combination of energy-dispersive spectroscopy, X-ray photoelectron spectroscopy, X-ray diffraction, and attenuated total reflectance–Fourier transform infrared spectroscopy analyses. Finally, each oxide group showed sustained Ca, P, and Mg ion release during an inductively coupled plasma spectroscopy dissolution assessment, and demonstrated early apatite-forming ability during simulated body fluid bioactivity testing. The findings of this study show much promise for the applicability of these novel oxide coatings to a wide variety of future titanium implant applications.

## 1. Introduction

With increasing life expectancy and an aging global population, improving the long-term performance of medical implants has become a priority. Titanium and its alloys have a long history of successful use for orthopedic and dental applications due to their excellent corrosion resistance, mechanical performance, and biocompatibility [1,2,3]. However, titanium alloys exhibit higher elastic moduli compared to natural bone [4,5,6,7,8]. The modulus mismatch between the implant alloys and the surrounding bone may lead to stress shielding and subsequent bone resorption. Additive manufacturing enables the production of porous lattice structures of titanium alloys with elastic moduli much closer to those of natural bone [6,9,10]. The porous lattice structures consist of repeating and periodically arranged unit cells with a defined geometry [6,9,10]. In addition to the modulus reduction, the 3D-printed porous lattice designs also promote bone ingrowth into the device to improve mechanical bone interlocking [6,11,12,13]. 3D-printed triply periodic minimal surfaces (TPMS) are surfaces with zero mean curvature and are periodic in three independent directions [14,15,16]. TPMS lattices can be tailored to match the moduli of natural bone while exhibiting superior compressive strengths [17]. Gyroid lattices are a subtype of TPMS lattice designs with unit cell structures that exhibit surface curvatures similar to those of trabecular bone and have been shown to improve bone fixation [6,9,16,17].

Even in well-made implants, aseptic loosening remains a challenge [1]. Aseptic loosening is a non-infectious failure mechanism that arises from inadequate mechanical fixation at the bone–implant interface [1]. Both micro-motion and wear particle accumulation may contribute to aseptic loosening. A number of physicochemical surface modification approaches have been previously implemented to improve the lifetimes of titanium implants by reducing aseptic loosening, strengthening interfacial bonding to bone, or accelerating osseointegration. Natural bone consists of 34.8 wt% calcium (Ca), 15.2 wt% phosphorus (P), and 0.72 wt% magnesium (Mg) [18,19]. These elements are actively involved in bone repair and regeneration [19,20]. Previously, Ca and P combination electro-deposition and magnetron sputtering coatings on titanium showed improved osteoblast cell attachment and coverage in vitro and enhanced bone growth and bone–implant contact in vivo [21,22]. Additionally, Arc-ion-plated Mg-doped titanium surfaces revealed superior cell adhesion, angiogenesis, and osteogenic differentiation when compared to uncoated titanium substrates [23]. Hydroxyapatite, a calcium phosphate phase, has long been used as a coating to functionalize implant surfaces due to its chemical similarity to bone tissue [24]. Conventional plasma-sprayed hydroxyapatite coatings are FDA-approved, but have been associated with numerous complications over the years [1]. Some noted complications include phase instability, reduced coating crystallinity, and limited interfacial adhesion resulting from thermal expansion differences between the hydroxyapatite and the non-coated titanium substrate [25,26]. Hydroxyapatite atomic crystalline lattice structures exhibiting carbonate (CO_3_^2−^) ion substitutions in place of hydroxyl or phosphate groups are referred to as biological apatite or bone-like apatite. These CO_3_^2−^ substitutions enhance ion exchange and solubility of hydroxyapatite, and have been shown to improve resorption rates [27,28].

Titanium implant surfaces, when exposed to oxygenated environments, spontaneously form a thin amorphous oxide layer that is inert and has physicochemical properties that are less than ideal for bone–implant surface interaction [2,3,29]. Anodization is a common electrochemical surface modification technique that can simultaneously produce complex micro- and nano-scaled oxide surface topographies, incorporate beneficial oxide surface chemistries, and crystallize titanium oxides into the more bioactive anatase and rutile phases [2,3,29]. Recent anodization strategies have focused on the use of customized electrolytes to incorporate elements such as Ca, P, or Mg, which are found in natural bone [2,3,26,29,30,31,32,33]. Anodization processes have also been utilized to incorporate calcium phosphate compounds, including hydroxyapatite, into the oxide coatings [2,25,26,30]. Anodization studies incorporating hydroxyapatite have shown enhanced coating–substrate adhesion due to the formation of a thin calcium titanate interlayer [25,26].

Recently, we have developed novel single-step anodization strategies using either natural or synthetic organic-acid-based electrolytes with Ca, P, and Mg sources to produce oxide coatings on titanium that mimic the composition of natural bone [34,35,36,37,38]. The oxide coatings were shown to incorporate Ca, P, and Mg bone elements and combinations of carbonated apatite and tricalcium phosphate compounds. The primary objective of the present study was to compare and contrast the surface characteristics of these novel anodized oxide coatings produced on solid and 3D-printed titanium alloy substrates. Our results point to potential applicability of these novel oxide coatings toward a wide range of clinical implant applications.

## 2. Materials and Methods

### 2.1. Specimen Preparation and 3D Printing Build Parameters

Solid disk specimens sectioned from Ti-6Al-4V ELI—a titanium alloy containing approximately 6 wt% aluminum and 4 wt% vanadium, extra-low interstitial grade (TAV), sourced from 12.7 mm diameter wrought bar stock (Fort Wayne Metals, Fort Wayne, IN, USA)—were sliced into 3 mm thick disk specimens (Accutom, Struers, Cleveland, OH, USA). Solid disk specimens were then ground with 220-grit SiC paper to remove rough edges created during sectioning. 3D-printed porous gyroid and diamond lattice disk specimens were fabricated using a - laser powder bed fusion (L-PBF) system (Renishaw AM 250, Wotton-under-Edge, Gloucestershire, United Kingdom) with TAV powder supplied by Carpenter Additive (Carpenter Technology Corporation, Philadelphia, PA, USA) with a particle size distribution of 15–45 µm. The gyroid specimen fabrication parameters were set to a 30% fill ratio with a 2 mm unit cell length to produce a 0.19 mm surface thickness. The diamond specimen fabrication parameters were set to a 30% fill ratio, a 1.92 mm unit cell length, and a 0.5 mm thickness. The fabrication process for both the gyroid and diamond 3D disks was carried out using a meandering scan strategy with the following scan parameters: laser power set to 200 W, scanning speed set to 1250 mm/s, layer thickness set to 30 μm, rotation angle set to 67°, and hatch distance set to 0.065 mm. The resulting gyroid and diamond disks produced by this process had a 10 mm diameter and a 2 mm thickness. Prior to testing, the solid, 3D-printed gyroid and 3D-printed diamond TAV alloy disks were cleaned in an ultrasonic bath with laboratory detergent (Alconox^®^, White Plains, NY, USA) for 30 min, followed by a rinse in distilled water for 30 s.

### 2.2. Disk Specimen Surface Characterization

The surface topography of the solid and 3D-printed specimens was captured using optical microscopy and laser-assisted 3D profilometry (LOP, Keyence, Osaka, Japan, VK-X3000). Surface roughness measurements (n = 3) were performed on the macroscopic LOP images of each disk specimen. Average surface roughness (S_a_) values and peak-to-valley roughness (S_z_) values were calculated for each of the solid and 3D-printed titanium substrates. Representative disk specimens from each group were mounted in conductive epoxy (Polyfast, Struers, Cleveland, OH, USA) and rotary-polished to a 0.05 µm surface finish in colloidal silica suspension (Struers, Cleveland, OH, USA). These polished samples were then vibratory-polished to a 0.02 µm surface finish in colloidal silica suspension (VibroMet 2, Buehler, Lake Bluff, IL, USA). Electron back-scattered diffraction (EBSD, EDAX, Mahwah, NJ, USA) analyses were utilized to generate microstructural inverse pole figure maps, phase distribution maps, and kernel average mismatch (KAM) residual strain maps for the solid and 3D-printed disk substrate materials.

### 2.3. Anodization Processing

The solid and 3D-printed disks were then submerged in a 10:1 ratio of nitric acid and hydrofluoric acid (TURCO NITRADD, Henkel Corporation, Madison Heights, MI, USA) for 30 s to activate the disk surfaces for anodization and rinsed in distilled water prior to being immersed in the anodization electrolyte. The anodization cell setup consisted of two CPTi strips as cathodes, and either a solid or 3D-printed TAV specimen disk as an anode submerged in the electrolyte. The anodization electrolyte was composed of citric acid (99+%, Fisher Scientific, Waltham, MA, USA), magnesium phosphate dibasic trihydrate (96.0%, Spectrum Chemical, New Brunswick, NJ, USA), and calcium acetate (99%, Spectrum Chemical, New Brunswick, NJ, USA) in distilled water in the concentrations provided in Table 1. Citric acid was selected as the acid component for this anodization electrolyte based on our previous anodization studies showing substantial incorporation of Ca, P, and Mg, as well as the formation of carbonated bone-like apatite within the oxides [37]. Magnesium phosphate and calcium acetate were selected to serve as Mg, P, and Ca sources in the electrolyte based on previous studies [34,35,36,37]. Pulsed galvanostatic anodization waveforms (700 mA/cm^2^ current density, 28% duty cycle, 7.2 Hz frequency, 120 s duration) were applied using a DC rectifier (350 V, 10 A, Dynatronix, Amery, WI, USA). Multiple disk specimens (n = 3) were anodized for each solid and 3D-printed disk specimen type. Post anodization, the disks were rinsed with distilled water and dried using clean, dry, and oil-free compressed laboratory air.

### 2.4. Anodized Oxide Surface Characterization

#### 2.4.1. Oxide Surface Topography

The surface topography of the solid and 3D-printed specimens was captured using LOP (Keyence, Osaka, Japan, VK-X3000) and scanning electron microscopy (SEM, Supra 40, Zeiss, Jena, Germany). Macroscopic images of each solid and 3D-printed disk specimen were captured using optical microscopy with the LOP microscope. Laser-assisted surface roughness measurements (n = 3) were also performed on the macroscopic LOP images of each disk specimen. Average surface roughness (S_a_) values and peak-to-valley roughness (S_z_) values were calculated for each group. SEM was used to capture the micro- and nano-scaled oxide surface topographies of each disk specimen.

#### 2.4.2. Oxide Surface Crystallinity

The crystallinity of the oxide layers on each group was assessed using thin-film X-ray diffraction (XRD; XDS 2000, Scintag, Franklin, MA, USA). XRD scans were conducted over a 2θ range of 24° to 42° using Cu-Kα radiation (λ = 0.154 nm), with specimens rotated 1° to verify the crystalline phases present within the anodized oxide layers.

#### 2.4.3. Oxide Surface Chemistry

The surface oxide elemental compositions from each oxide group were determined using a combination of energy-dispersive X-ray spectroscopy (EDS; EDAX APEX, Mahwah, NJ, USA) and X-ray photoelectron spectroscopy (XPS; Thermo Scientific K-Alpha, Waltham, MA, USA). Triplicate EDS spectra were collected from different areas on each anodized disk specimen at 500× magnification using an accelerating voltage of 12 kV. The average elemental concentrations of Ca, P, and Mg, and the corresponding surface Ca/P ratios, were calculated for each oxide group. Representative specimens from each oxide group were also analyzed with XPS to determine the chemical compositions of the outermost oxide surfaces. For XPS, a monochromatic Al Kα X-ray source (1486.6 eV) was used at 75 W and 12 kV, with a spot size of 400 μm^2^. Calibration was performed using the Au 4f_7_/_2_ (84.0 eV) and C1s (284.8 eV) peaks. XPS survey scans were collected at a pass energy of 200 eV with a 1.0 eV step size. High-resolution XPS spectra were then captured to compare the C_1s_, O_1s_, P_2p_, Mg_1s_, Mg_2p_, and Ca_2p_ levels present in each oxide group using a 40 eV pass energy and a 0.1 eV step size, averaged over 50 scans.

#### 2.4.4. Oxide Surface Molecular Structure

The molecular structures present in each oxide group were also assessed with attenuated total reflectance–Fourier transform infrared spectroscopy (ATR-FTIR; Spectrum 100, Perkin-Elmer, Waltham, MA, USA), scanning a spectral range from 650 to 4000 cm^−1^ at a spectral resolution of 4 cm^−1^.

### 2.5. Oxide Coating Dissolution Assessment

An oxide coating dissolution study was performed to measure the Ca^2+^, Mg^2+^, and PO_4_^3−^ ion release profiles from the anodized oxides on solid and 3D-printed titanium substrates. For this experiment, anodized disk specimens (n = 3) of each type were immersed in 10 mL of distilled de-ionized water in 50 mL centrifuge tubes. The anodized specimens were transferred to new centrifuge tubes with 10 mL of fresh distilled water at specific study time points of 1, 3, 5, and 7 days. Any remaining particulate matter in each collected solution was dissolved by adding 300 μL of nitric acid and 50 μL of hydrochloric acid, followed by filtration using 0.22 μm syringe filters. The filtered solutions were then analyzed for ion release concentrations in parts per million (ppm) using inductively coupled plasma optical emission spectrometry (ICP-OES, SPECTRO AMETEK, SPECTROGREEN, Kleve, Germany, SPECTRO ICP Analyzer Pro Software version 1.30.0062).

### 2.6. Oxide Coating Bioactivity Assessment

Simulated body fluid (SBF) was prepared according to ISO Standard 23317 [39]. Anodized specimens (n = 3) from each group were submerged in SBF for a period of 7 days at 37 °C. After SBF soaking, specimens were gently rinsed with distilled water and dried in a desiccator at room temperature for at least 16 h. XRD scans were then conducted over a 2θ range of 28° to 34° to assess the formation of any additional apatite on the anodized oxide surfaces on the solid and 3D-printed titanium disk substrate materials.

### 2.7. Statistical Analysis

Multiple unpaired t-tests (*α *= 0.05) were used to evaluate statistical differences in the surface roughness values between non-anodized and anodized specimens within each group. A two-way ANOVA (*α *= 0.05) with a post hoc Tukey’s multiple comparisons test was used to assess the influence of titanium alloy substrate type on dopant element uptake in the anodized coatings. One-way ANOVA (*α *= 0.05) with post hoc Tukey’s analyses were applied to determine any significant differences in the Ca/P ratios between the oxides and to determine differences between metal ion release levels from the oxide groups at day 1 and day 7.

## 3. Results and Discussion

### 3.1. Specimen Surface Characteristics

**Figure 1:**
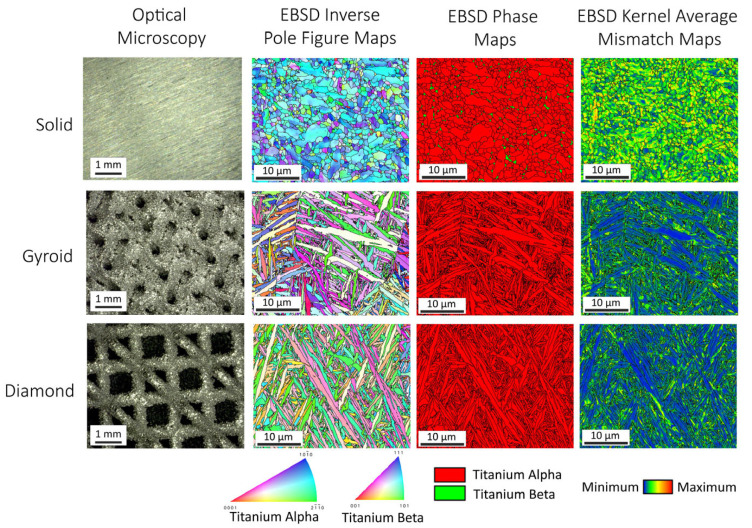
Microstructure and crystallographic analysis for the solid and 3D-printed lattice non-anodized substrate groups. The leftmost column contains optical microscopy images. The second and third columns provide electron back-scattered diffraction (EBSD) inverse pole figure and phase maps. The rightmost column provides the EBSD 4th order kernel average mismatch (KAM) maps. Higher residual strain levels were shown in the solid substrate KAM maps compared to those of the 3D-printed substrates.

Figure 1 provides the surface appearances and microstructural analyses of non-anodized specimens from the solid and 3D-printed titanium alloy substrate materials. The leftmost column of Figure 1 provides optical microscopy images captured using the LOP. The solid disk specimens still showed surface grooves from the disk sectioning process. In contrast, the 3D-printed gyroid and diamond disk specimens revealed macroscopic porous lattice structures produced by the L-PBF printing processes. The 3D-printed gyroid lattice specimens exhibited macroscopic porosity, with mean pore surface areas of 0.17 ± 0.09 mm^2^. The 3D-printed diamond lattices revealed comparatively larger open square-shaped pores with mean pore surface areas of 0.62 ± 0.18 mm^2^. Both the gyroid and diamond group specimens revealed a rough surface appearance produced by remaining TAV powder particles sintered to the outer surfaces.

EBSD inverse pole figure maps and phase maps of representative areas of polished specimens from each substrate group are provided in the second and third columns from the left in Figure 1. For the solid disk specimens, the average alpha phase grain size was 1.7 ± 1.0 μm and the average beta grain size was 0.4 ± 0.2 μm. The solid specimen EBSD phase maps revealed 96.8% alpha phase grains and 3.2% beta phase grains. In contrast, the 3D-printed gyroid and diamond disk specimens revealed a lamellar alpha martensitic phase microstructure. The formation of this lamellar alpha martensitic phase microstructure was due to the rapid cooling processes involved in L-PBF specimen fabrication. Specifically, for the 3D-printed gyroid disk specimens, the alpha phase laths exhibited an average major axis lath length of 5.3 μm and an average minor axis lath width of 1.2 μm. The 3D-printed diamond lattice specimens also showed a similar lamellar alpha martensitic phase microstructure, with an average major axis lath length of 6.2 μm and an average minor axis lath width of 1.1 μm. The EBSD phase maps revealed that no beta phase was detected in either of the 3D-printed disk specimen substrates. Thus, the microstructures for the solid and 3D-printed titanium alloy substrates in the present study were fundamentally different, as shown in Figure 1.

The rightmost column of Figure 1 provides fourth-order kernel average mismatch (KAM) maps for the solid and 3D-printed groups. These KAM maps allow us to qualitatively visualize the relative residual strain levels in each substrate material. In KAM maps, residual strain levels are color-coded, with blue areas representing the lowest levels. Increasing strain levels are depicted with green, yellow, orange, and red colors. Qualitatively, the solid disk specimens exhibited higher residual strain levels, as indicated by the green-to-yellow-colored KAM maps. This finding was not surprising given that the solid disk specimens were sectioned from drawn wrought bar stock. In contrast, the 3D-printed gyroid and diamond substrates, which exhibited the martensitic lamellar alpha phase microstructures, revealed lower residual strain with blue-to-green-colored KAM maps. Previous anodization studies on aluminum alloy substrates have noted that oxides formed on substrates with cold-worked microstructures, with higher residual strain or dislocation density, exhibited different surface topographies and coating adhesion properties compared to those formed on annealed substrates [40,41].

### 3.2. Anodized Surface Characteristics

#### 3.2.1. Oxide Surface Topography Assessment

**Figure 2:**
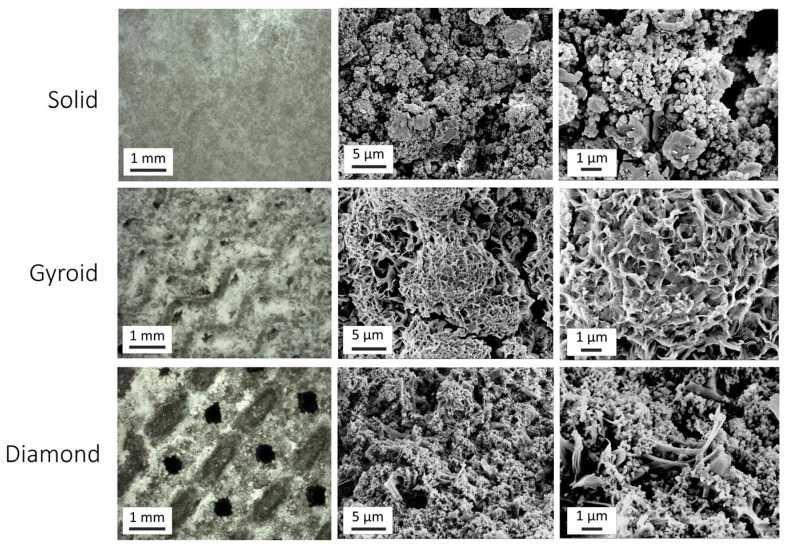
An oxide surface topography comparison. The leftmost column displays optical microscopy images. The 3D-printed oxides retained some of the macroscopic surface porosity after anodization. The second and third columns show micro- and nano-scaled scanning electron microscopy images of each group. The solid and 3D-printed diamond specimen groups revealed a granular oxide surface topography on the micro and nano scales. The diamond specimen oxides also showed small clusters of petal-like features. In contrast, the gyroid specimen oxides revealed aggregated petal-like features, with unique rosette pattern formations.

Figure 2 compiles the anodized specimen surface topographies for each group. Macro-scale optical microscopy images for each group are shown in the leftmost column. The solid oxide specimen appears dense and completely covers the sectioning grooves, which were visible in the corresponding non-anodized substrate images in Figure 1. The 3D-printed gyroid and diamond group oxides showed macroscopic porosity patterns similar to those of their corresponding non-anodized substrate images in Figure 1. After anodization, the remaining average surface areas of the macroscopic pores on the 3D-printed substrates were 0.11 ± 0.04 mm^2^ for the gyroid specimens and 0.30 ± 0.16 mm^2^ for the diamond specimens. The micro- and nano-scale surface topographies of the oxides are shown in the middle and rightmost columns, respectively. The solid specimen exhibited a granular oxide pattern at both scales. The 3D-printed diamond specimen oxide also showed a granular oxide pattern at the micro scale with patches of petal-like features at the nano scale. In contrast, the 3D-printed gyroid disk specimens revealed micro- and nano-scale topographies consisting of aggregated petal-like features, forming a unique rosette-like pattern. The granular oxide pattern observed on solid and 3D-printed diamond disk oxide surfaces has been previously reported in our Mg-doped hydroxyapatite-containing oxides produced using similar electrolyte chemistries [34,36,37]. The petal-like features were also previously observed in our hydroxyapatite-containing oxides produced using citrus-juice-based electrolytes [34,36,37]. To our knowledge, the rosette pattern clusters of aggregated petal-like features shown in the 3D-printed gyroid disk specimens are unique and have not been shown before.

**Figure 3:**
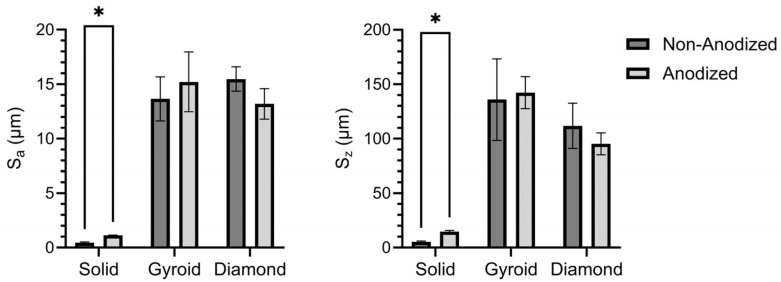
Surface roughness analysis on each solid or 3D-printed group using laser-assisted 3D profilometry. (**left side figure**) Average surface roughness (S_a_) and (**right side figure**) peak-to-valley height (S_z_). Anodization resulted in a significant increase in both S_a_ and S_z_ values for the solid specimen group (*p* < 0.05). In contrast, anodization did not significantly alter the surface roughness on the 3D-printed gyroid and diamond disk specimens. * symbol indicates statistically different groups with a significance level of *p* < 0.05.

Figure 3 compares the average surface roughness (S_a_) values in Figure 3 (left side) and peak-to-valley height (S_z_) values in Figure 3 (right side) between the non-anodized and anodized counterparts from each substrate group. Anodization resulted in a significant increase in both the S_a_ and S_z_ values for the solid specimen group (*p* < 0.05). Specifically, the non-anodized solid specimens showed average surface roughness (S_a_) values of 0.44 ± 0.09 μm, whereas the anodized solid specimens showed higher S_a_ values of 1.13 ± 0.03 μm. In contrast, the non-anodized 3D-printed disk specimen substrates exhibited macroscopic roughness due to the laser powder bed fusion additive manufacturing process. The non-anodized 3D-printed gyroid specimens revealed S_a_ values of 13.66 ± 2.02 μm, and the non-anodized 3D-printed diamond specimens revealed S_a_ values of 15.48 ± 1.13 μm. The additional micro-scale roughness introduced during the anodization processes did not significantly alter the overall macro-scale surface roughness observed on the 3D-printed substrates. Specifically, the anodized 3D-printed gyroid specimens showed S_a_ values of 15.21 ± 1.25 μm, and the anodized 3D-printed diamond specimens showed S_a_ values of 13.19 ± 1.39 μm. Previous studies have shown micro-scaled roughness profiles to enhance bone–implant contact, strengthen bone-to-implant surface adhesion, and produce more predictable long-term clinical outcomes [42]. In addition to the micro- and macro-roughness levels shown in the surface roughness analyses, the solid and 3D-printed groups also revealed complex nano-scaled surface topographies nested within these micro- and macro-rough surface profiles as shown in the topographical SEM analyses in Figure 2. Nano-scaled roughness profiles on titanium oxide layers have previously been shown to improve protein adsorption and increase osteoblast cell attachment and differentiation [43,44].

#### 3.2.2. Oxide Crystallinity Analyses

**Figure 4:**
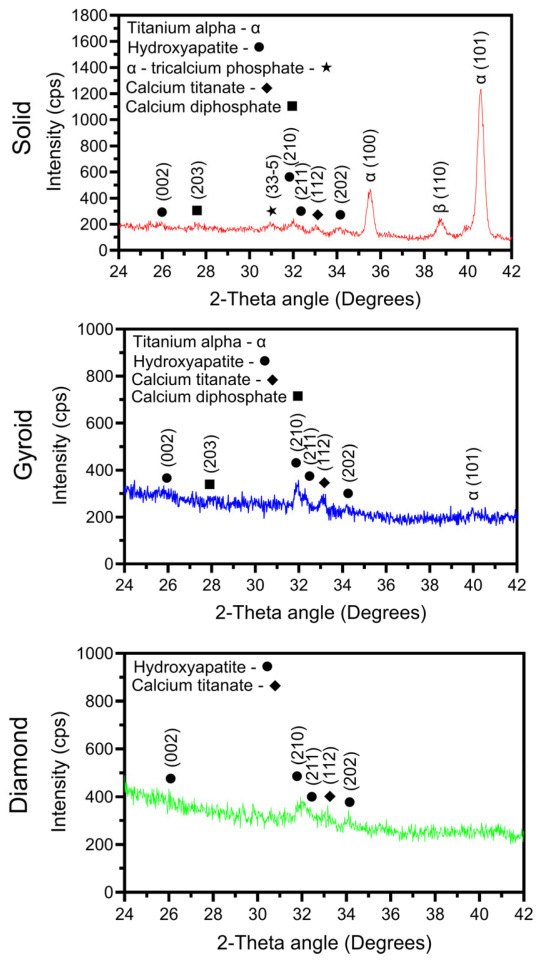
Representative X-ray diffraction scans from each anodized group. The solid disk group exhibited diffraction peaks for hydroxyapatite, α-tricalcium phosphate, calcium diphosphate, and calcium titanate, along with α-phase and β-phase titanium peaks. The 3D-printed gyroid specimen disk oxides exhibited diffraction peaks for hydroxyapatite, calcium titanate, and calcium diphosphate formation. A small α-phase titanium peak was also observed on the gyroid group, which indicated a relatively thicker surface oxide layer formation. The 3D-printed diamond group oxides showed hydroxyapatite and calcium titanate phase diffraction peaks, with no visible α-phase or β-titanium phase titanium peaks. Thus, a thicker oxide layer was also formed on the 3D-printed diamond group.

Figure 4 compiles X-ray diffraction scans from each oxide group. The solid specimen oxide group exhibited diffraction peaks for hydroxyapatite, α-tricalcium phosphate, calcium diphosphate, and calcium titanate, along with α- and β-titanium phase peak contributions from the underlying titanium substrate material. The presence of significant titanium peaks in the solid specimen XRD spectra indicated the presence of a thin surface oxide layer. The 3D-printed gyroid oxide group, in contrast, exhibited hydroxyapatite, calcium titanate, and calcium diphosphate phase peaks, along with a comparatively smaller α-phase titanium peak indicative of thicker oxide layer formation. No β-phase titanium diffraction peaks were observed, which agrees well with the EBSD microstructural phase analyses shown in Figure 1. The 3D-printed diamond oxide group revealed hydroxyapatite and calcium titanate phase diffraction peaks, with no evidence of α-phase or β-phase titanium peaks. Thus, both 3D-printed lattices formed thicker oxide layers compared to the solid wrought titanium substrate materials. The formation of these thicker oxide layers is likely attributable to a combination of the differences in surface areas of the solid and 3D-printed disk specimens exposed to the anodization electrolyte, the differences shown in the solid and 3D-printed substrate microstructures (Figure 1) and the differences in the residual strain levels shown between the solid and 3D-printed substrate materials (Figure 1). Additionally, it should be noted that the petal-like morphological features observed in the surface topographies of the 3D-printed groups in Figure 2 have previously been associated with hydroxyapatite phase formation [3,30,36]. Thus, the SEM and XRD analysis findings in the present study are in good agreement. In addition to hydroxyapatite, all the oxides show the formation of calcium titanate. Previous studies have shown that calcium titanate formation during anodization improves mechanical adhesion between the oxide coating and the titanium substrate [25,26].

#### 3.2.3. Oxide Surface Compositions

**Figure 5:**
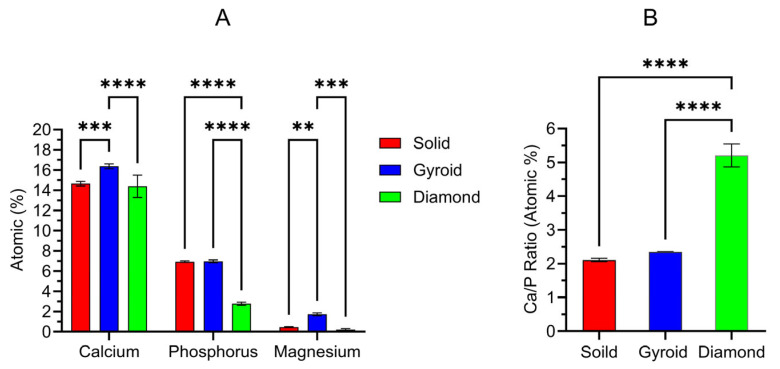
Energy-dispersive X-ray spectroscopy-derived surface compositions from each anodized oxide group. (**A**) Relative Ca, P, and Mg dopant uptake levels incorporated into each oxide group and (**B**) relative Ca/P ratios calculated for each oxide group. ** symbols indicate statistically different groups with a significance level of *p* < 0.01. *** symbols indicate statistically different groups with a significance level of *p* < 0.001. **** symbols indicate statistically different groups with a significance level of *p* < 0.0001.

Figure 5 provides the surface-derived EDS results from each anodized oxide group. Figure 5A shows the relative Ca, P, and Mg dopant uptake levels into each oxide. The corresponding surface Ca/P ratios are presented in Figure 5B. The two-way ANOVA (α = 0.05) analyses of the EDS surface chemistries revealed a significant interaction between the titanium disk substrate type and the oxide dopant element uptake type (*p* < 0.0001). The main-effects analyses showed significant effects for both the titanium disk substrate type and the oxide dopant element uptake type (*p* < 0.0001). Post hoc Tukey comparisons revealed that the 3D-printed gyroid disk substrates exhibited significantly higher (*p* < 0.0005) Ca dopant uptake levels of 16.4 ± 0.2 at% compared to the solid and 3D-printed diamond disk oxides with dopant levels of 14.7 ± 0.2 at% and 14.4 ± 0.9 at%, respectively. The solid and 3D-printed gyroid group oxides exhibited statistically similar P or phosphate dopant uptake levels of 6.9 ± 0.0 at% and 7 ± 0.1 at%. In contrast, the 3D-printed diamond group oxides showed significantly lower (*p* < 0.0001) P or phosphate dopant uptake levels of 2.8 ± 0.1 at%. The 3D-printed gyroid group oxides also showed the highest Mg dopant uptake levels of 1.7 ± 0.1 at%, which were significantly higher (*p* < 0.01) than the solid and 3D-printed diamond group oxide levels of 0.5 ± 0.0 at% and 0.2 ± 0.1 at%. It should also be mentioned that the high Mg dopant uptake levels in the 3D-printed gyroid group oxides represent the highest Mg dopant levels we have observed in our organic-acid-based anodization studies, where hydroxyapatite was also formed within the oxide layers [37,38]. Previously, Mg dopant uptake levels of 1.4 ± 0.1 at% were shown for oxides produced in natural citrus-juice-based electrolytes [34]. Notably, we have observed even higher Mg dopant uptake levels of 5.0 ± 0.0 at% in our oxalic-acid-based anodization studies, but the resulting oxides did not show hydroxyapatite phase formation in the XRD analyses [38]. Overall, the differences shown in the Ca, P, and Mg uptake levels for the oxides in the present study formed on different titanium substrate types are likely attributable to a combination of the differences in the surface areas of the solid and 3D-printed specimens exposed to anodization, the differences in the substrate material microstructures as shown in Figure 1, and the differences in the substrate residual strain levels as shown in Figure 1.

As a result of the low P dopant uptake levels, the 3D-printed diamond group oxides also revealed the highest EDS-derived surface Ca/P ratios of 5.2 ± 0.3. In contrast, the solid and 3D-printed gyroid group oxides revealed significantly lower (*p *< 0.0001) Ca/P ratios of 2.1 ± 0.0 and 2.3 ± 0.0, respectively. The solid and 3D-printed gyroid group oxide surface Ca/P ratios are closer to the stoichiometric hydroxyapatite Ca/P ratio (1.67) [45].

**Figure 6:**
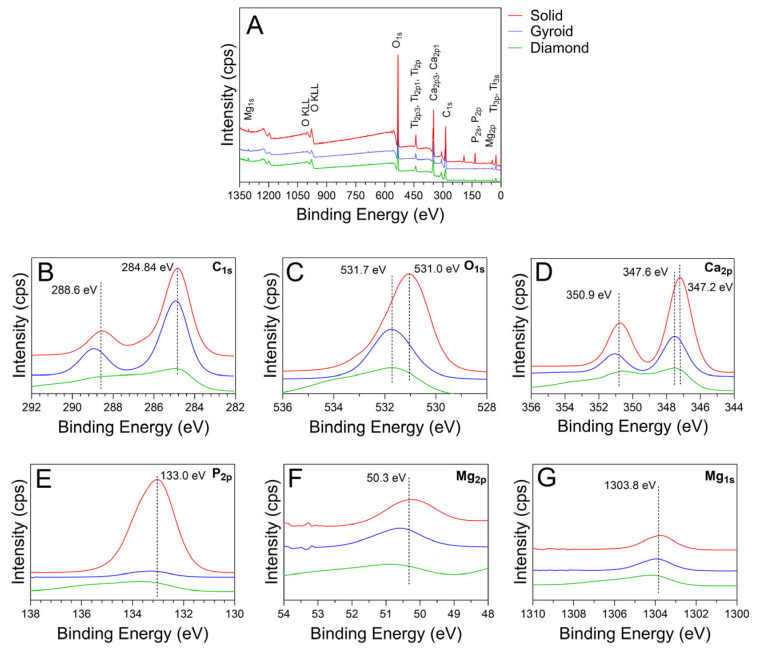
X-ray photoelectron spectroscopy (XPS) analysis of the outermost surface compositions for each anodized oxide group. (**A**) Representative XPS survey spectra for each oxide group showing peaks for O, Ca, P, Mg, and C. (**B**) High-resolution spectra showing C_1s_ peaks with binding energies of 284.84 eV and 288.6 eV were observed in all oxides. The C peaks were less prominent in the 3D-printed diamond group oxides. (**C**) High-resolution spectra showing O_1s_ peaks with a binding energy of 531.0 eV were present for the solid group oxides, while in the 3D-printed group, O_1s_ peak positions revealed a minor shift to a higher binding energy of 531.7 eV. These O peaks represent calcium phosphate-containing oxides. (**D**) High-resolution spectra showing a Ca2p band with well-resolved doublet peak components, Ca2p3/2 and Ca2p1/2, were observed for all oxide groups. These Ca peaks represent the formation of calcium titanate and calcium phosphates. For the solid group oxides, these peaks were located at 347.2 eV and 350.9 eV, while for the 3D-printed group oxides, the Ca_2p3/2_ component peak showed a shift to a higher binding energy of 347.7 eV. (**E**) High-resolution spectra showing P_2p_ peaks with a binding energy of 133 eV were observed in all oxides. These P peaks indicated the presence of phosphorus-containing titanium oxide and phosphate formation. (**F**) High-resolution spectra showing Mg_2p_ at 50.3 eV in each oxide. These Mg peaks have been attributed to the formation of Mg-doped hydroxyapatite. (**G**) High-resolution spectra showing Mg_1s_ peaks at 1303.8 eV in each oxide. This Mg peak has been attributed to the formation of Mg-containing oxides.

Representative XPS spectra for each anodized oxide group are provided in Figure 6. The XPS survey spectra for all the oxide groups show peaks for O, Ca, P, Mg, Ti, and C, as shown in Figure 6A. The XPS-derived peak intensities for all elements were substantially lower for the 3D-printed gyroid and diamond group oxides compared to the solid disk oxide. The differences in intensities shown are likely attributable to the differences in the surface topographies of the three substrate groups. The sectioned solid group surfaces provided a flat and even surface for XPS sampling that provided good intensity values. In contrast, the complex and continuously varying surface topographies of the porous 3D-printed gyroid and diamond group surfaces likely showed somewhat reduced X-ray signals reflected from the complex surfaces, and thus relatively reduced the peak intensities. High-resolution C_1s_ peaks having binding energies of 284.84 eV and 288.6 eV, as shown in Figure 6B, were found to be prominent in the solid and gyroid group oxides and less prominent in the diamond group oxides. These peaks represent the presence of C-C bonds and carbonates in the oxides [43,46]. The C_1s_ peak at 284.84 eV binding energy also represents surface contamination carbon [47,48]. Figure 6C shows high-resolution spectra for the O_1s_ peaks with a binding energy of 531.0 eV for the solid group oxides. Notably, 3D-printed gyroid and diamond group oxides revealed a minor shift to a higher O_1s_ peak binding energy of 531.7 eV. These O_1s_ peak positions represent the presence of calcium phosphate-containing oxides on the outermost surfaces of the anodized solid and 3D-printed groups [46,49]. Each anodized oxide group showed a Ca_2p_ band with well-resolved doublet peak components Ca_2p3/2_ and Ca_2p1/2_, as shown in Figure 6D. These Ca peaks represent the formation of calcium titanate and calcium phosphates found in these oxides, as observed in the corresponding XRD datasets in Figure 4 [43,46,49,50,51,52,53,54]. For the solid group oxides, these peaks were located at 347.2 eV and 350.9 eV. In contrast, for the 3D-printed gyroid and diamond group oxides, the Ca_2p3/2_ component peak showed a shift to a higher binding energy of 347.7 eV. Interestingly, such shifts to higher binding energies have previously been shown to indicate an increase in the amount of crystalline hydroxyapatite formed [54]. This finding agrees well with the higher hydroxyapatite phase peak intensities shown in the XRD analyses for the anodized 3D-printed groups in Figure 4. However, it should be noted that the XPS-derived Ca peak intensities, indicative of the outermost oxide layer composition, were substantially lower for the 3D-printed gyroid and diamond group oxides as compared to the solid group oxides. A P_2p_ peak, with a binding energy of 133.0 eV, was found in all oxides, as shown in Figure 6E. This P peak is indicative of the formation of phosphorus-containing titanium oxides and phosphates [43,46,50]. The XPS-derived P peak intensities were also substantially lower for the 3D-printed group oxides in comparison to those for the solid group oxides. These findings indicate that the Ca and P compositions within the formed outermost surfaces of the 3D-printed group oxides were different to those of the solid group oxide counterparts.

Each oxide group also revealed peaks for Mg_2p_ and Mg_1s_, as shown in Figure 6F,G. These Mg peaks showed binding energies of 50.3 and 1303.8 eV, and are representative of the formation of Mg-containing oxides [55]. Interestingly, the presence of the Mg_2p_ peak has previously been associated with bonding between the Mg^2+^ and PO_4_^2−^ groups and is commonly observed in magnesium-doped hydroxyapatite [56]. Thus, the presence of the Mg_2p_ peak in the anodized oxides in the present study suggests that at least some of the Mg oxide doping present functions as a lattice substitution within the hydroxyapatite structure. This finding indicates the formation of a Mg-doped hydroxyapatite within our anodized oxides. Previously, a hydrothermal synthesis study that produced Mg-doped hydroxyapatite showed improvements in the material’s compressive strength and fracture toughness with increased Mg doping levels [57].

#### 3.2.4. Oxide Molecular Structure Analyses

**Figure 7:**
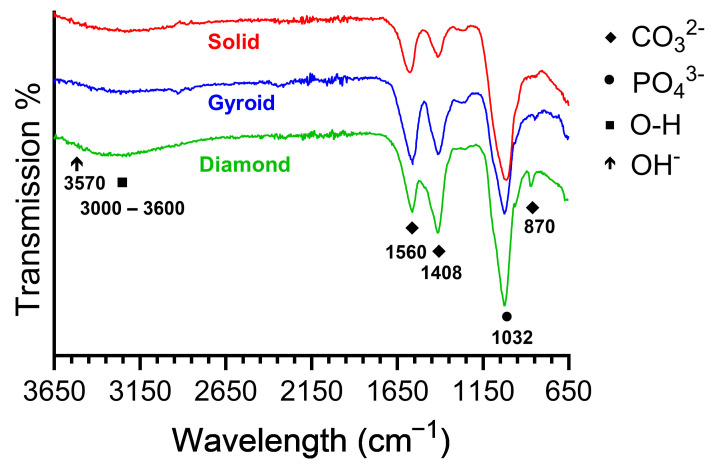
Representative Fourier transform infrared spectroscopy spectra for each oxide group. Each oxide group showed strong PO_4_^3−^ absorption peaks at 1032 cm^−1^, indicative of the formation of calcium phosphate phases. Additionally, weak O-H bands were shown between 3000 and 3600 cm^−1^, corresponding to the bending mode of adsorbed water. A poorly defined characteristic hydroxyapatite OH- peak was also shown at 3570 cm^−1^. High-intensity CO_3_^2−^ substitution peaks at 870, 1408, and 1560 cm^−1^, indicative of carbonate substitutions into the apatite lattice, were also present in each oxide group. 3D-printed diamond group oxides show comparatively higher intensity of B-type substitutions compared to A-type apatite lattice substitutions. In contrast, solid and 3D-printed gyroid group oxides showed a relatively higher intensity of A-type compared to B-type substitutions. Nonetheless, each oxide group showed a combination of AB-type carbonated apatite characteristic of bone-like apatite formation.

Figure 7 compiles representative FTIR spectra for each oxide group. Each oxide group revealed a strong PO_4_^3−^ absorption peak at 1032 cm^−1^. This peak indicated the presence of phosphate groups such as di- and triphosphates and apatite within the anodized oxides. This finding also agrees well with the phosphate and apatite phase XRD peaks shown for each anodized oxide group in Figure 4. A weak O-H band is shown for each oxide group between 3000 and 3600 cm^−1^. This band represents the bending mode of adsorbed water. However, the characteristic hydroxyapatite OH^−^ peak at 3570 cm^−1^ was poorly defined for each of the oxides. Instead, each oxide group showed CO_3_^2−^ peaks at 870, 1408, and 1560 cm^−1^ [26,28,58,59]. This finding is in good agreement with the carbonate peaks shown in the outermost surface XPS analysis for each oxide, as shown in Figure 6B. The presence of these carbonate peaks instead of hydroxyapatite OH^−^ peaks indicates the presence of carbonate substitutions within the hydroxyapatite. Carbonate-substituted hydroxyapatite is a characteristic of bone-like apatite formation [26,58,59]. In the literature, two types of carbonate substitutions are explained: (a) A-type substitutions of OH^−^ with CO_3_^2−^ (1560 cm^−1^) and (b) B-type substitutions of PO_4_^3−^ with CO_3_^2−^ (1408 and 870 cm^−1^) [28]. A combination of A-type and B-type substitutions is commonly known as AB type [28]. Natural bone primarily consists of B-type carbonated apatite, with a relatively small fraction of A-type substitutions, showing an AB-type substituted lattice [28]. Carbonate substitutions within the apatite lattice have been known to increase apatite solubility, facilitating osteoclastic resorption and bone remodeling and promoting more bone reformation [28]. The 3D-printed diamond group oxides in the present study showed higher intensities of B-type substitutions compared to A-type substitutions. In contrast, the solid and 3D-printed gyroid group oxides showed relatively higher intensities of A-type substitutions compared to B-type substitutions. Nonetheless, each oxide group showed the presence of some combination of AB-type carbonated apatite in the anodized layers.

#### 3.2.5. Oxide Dissolution Analyses

**Figure 8:**
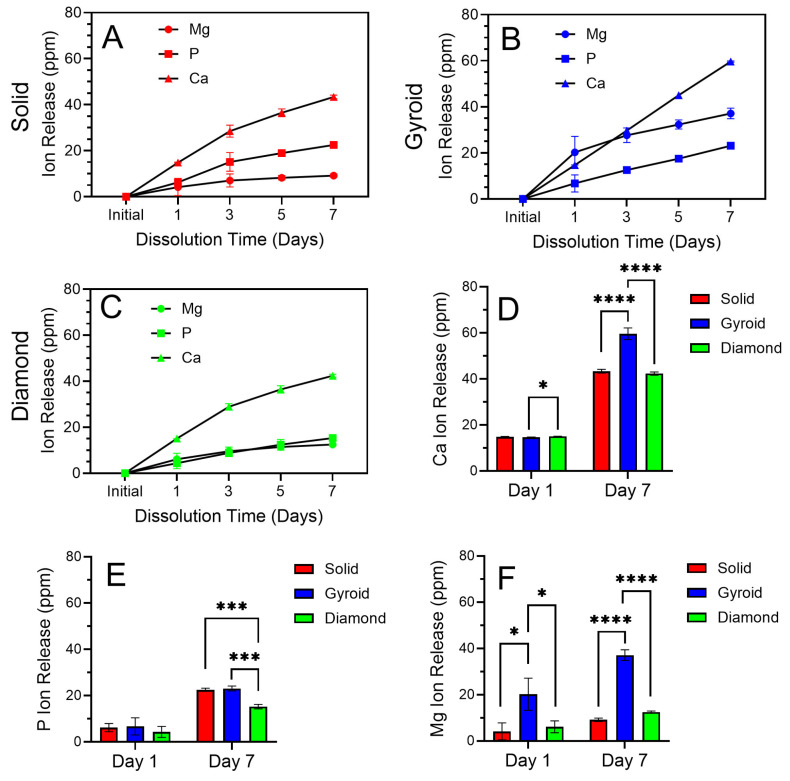
ICP-OES cumulative release profiles for Ca, P, and Mg ions from each oxide over 7 days in distilled water. (**A**) Cumulative 7-day Ca, P, and Mg ion release profiles from solid group oxides. (**B**) Cumulative 7-day Ca, P, and Mg ion release profiles from 3D-printed gyroid group oxides. (**C**) Cumulative 7-day Ca, P, and Mg ion release profiles from 3D-printed diamond group oxides. (**D**) Comparison of Ca ion release levels across oxide groups at day 1 and day 7. (**E**) Comparison of P ion release levels across oxide groups at day 1 and day 7. (**F**) Comparison of Mg ion release levels across oxide groups at day 1 and day 7. * symbol indicates statistically different groups with a significance level of *p* < 0.05. *** symbols indicate statistically different groups with a significance level of *p* < 0.001. **** symbols indicate statistically different groups with a significance level of *p* < 0.0001.

Cumulative ICP-OES-derived Ca, P, and Mg ion release profiles over a 7-day period from each oxide group are provided in Figure 8. Figure 8A–C provide the cumulative release profiles for Ca, P, and Mg over the 7-day duration. Each element showed a sustained release over the entire duration, with a somewhat higher burst release observed at the early day 1 timepoint. In general, the release of Ca ions was consistently higher than that of P and Mg for each oxide group over the test duration. The 3D-printed gyroid oxides, in Figure 8B, exhibited the highest cumulative ion release levels, particularly for Ca and Mg ions. The 3D-printed diamond oxides, in Figure 8C, showed comparatively lower release, particularly for P. Both of these ion release observations correlate very well with the findings from the EDS-derived surface composition analyses for these oxides shown in Figure 5. The presence of Ca and Mg ions in the implant microenvironment plays a critical role in enhancing osseointegration. Ca ions promote fibrin clot formation and osteogenic cell recruitment, supporting cell attachment, proliferation, and differentiation [60,61], while Mg ions are known to activate osteogenic signaling pathways such as TRPM7/PI3K and stabilize β-catenin during early differentiation stages [61]. Each of the oxide surfaces in the present study showed substantially higher Ca and Mg release, compared to a previous study on Ca- and Mg-doped titanium surfaces prepared using wet chemical treatments over similar timeframes [61]. In our previous anodization study using citrus-fruit-based electrolytes, the hydroxyapatite-containing oxides showed similar Ca ion release levels, but lower Mg ion release levels compared to the present study solid group oxides [36].

To further clarify the differences shown in ion release levels between the oxide groups in the present study, the day 1 burst ion release levels and the cumulative day 7 ion release levels for the Ca, P, and Mg ions from each oxide are also compiled separately in Figure 8D–F. One-way ANOVA (α = 0.05) with post hoc Tukey’s analyses was used to differentiate these results. The initial burst release levels observed at day 1 suggest the release of loosely bound ions on or near the oxide outermost surfaces. The Ca ion release at day 1 for the 3D-printed diamond group oxides was shown to be significantly higher than that of the 3D-printed gyroid group oxides (*p* = 0.0446). No significant differences were shown at day 1 in the P ion release. Mg ion release at day 1 was significantly higher for the 3D-printed gyroid group oxides compared to the solid group oxides (*p* = 0.0148) and the 3D-printed diamond group oxides (*p* = 0.0261). The substantially higher cumulative ion release levels shown at day 7 are indicative of sustained, gradual dissolution of the bulk oxide layers. Cumulative Ca ion release after day 7 was significantly higher for the 3D-printed gyroid group oxides compared to the solid group oxides and the 3D-printed diamond group oxides (*p* < 0.0001). Cumulative P ion release after day 7 was shown to be significantly higher for the solid group oxides (*p* = 0.0002) and 3D-printed gyroid group oxides (*p* = 0.0001) compared to the 3D-printed diamond group oxides. Finally, cumulative Mg ion release after day 7 was significantly higher for the 3D-printed gyroid group oxides compared to the solid group oxides and the 3D-printed diamond group oxides (*p* < 0.0001). The relatively higher Mg ion release observed for the gyroid group oxides may provide additional biological benefits by enhancing early osteogenic activity. Overall, the sustained Ca, P, and Mg ion release from these oxide groups suggests a strong potential to improve the biological performance of future implant coatings.

#### 3.2.6. Oxide Bioactivity Analyses

**Figure 9:**
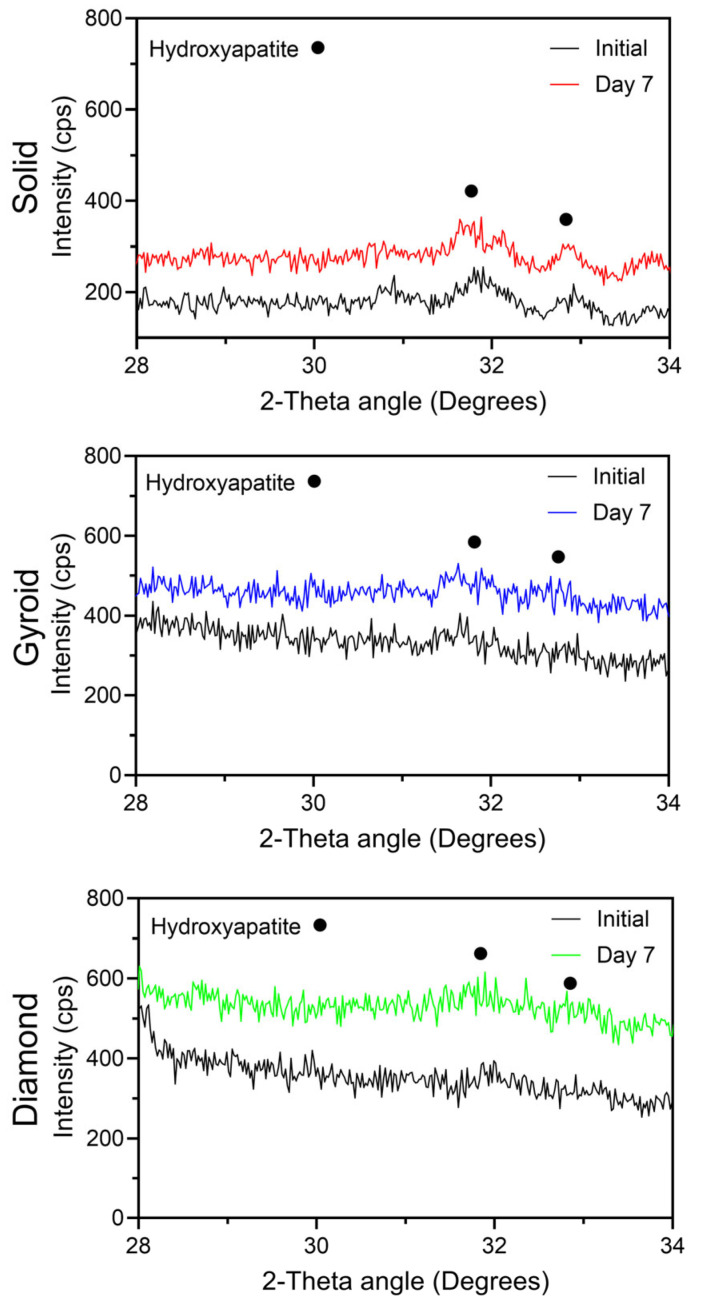
Representative X-ray diffraction scans from the 7-day SBF soak bioactivity assessment for each oxide group. Each oxide group showed a slight increase in hydroxyapatite peak intensities after the 7-day SBF soak period, indicating good early apatite-forming ability.

Figure 9 compiles the SBF soak bioactivity assessment results for each oxide group. For each oxide group, some increase in the hydroxyapatite XRD peak intensities was observed after the 7-day SBF soak period. These findings indicated each oxide group showed early apatite-forming ability. Previously, it has been shown that Ca^2+^ deposition and the formation of a phosphate layer at the implant–fluid interface are considered the initiating steps for bone-like apatite crystallization on bioactive surfaces [62]. It has also been shown that combining surface modification techniques with porous implant structures improves biocompatibility and osteogenic potential beyond either factor alone [63]. These findings from prior studies align well with the bioactivity performance of the 3D-printed anodized oxide groups from the present study.

The present study demonstrates that our novel citric-acid-based electrolyte anodization processes can be applied to create promising AB-type carbonated apatite-containing oxide layers on both solid and 3D-printed lattice-structured titanium alloy implants. However, some limitations of the study findings should also be noted. One recognized limitation is that the 3D-printed lattice disk specimens were anodized in their as-printed condition. Due to rapid cooling during laser powder bed fusion specimen fabrication, a predominantly alpha martensitic lath microstructure was produced for both 3D-printed substrate types. Thus, the microstructures of the titanium alloy substrates for the solid and 3D-printed specimen groups in the present study were fundamentally different, as shown in Figure 1. In future studies, it would be interesting to perform a controlled annealing heat treatment on the 3D-printed titanium lattice disk specimens to restore the duplex alpha–beta phase lattice structure prior to anodization. These additional specimen groups would provide a greater understanding of the effects of titanium alloy substrate microstructures on the oxide layer structures produced in these anodization processes. A second recognized limitation of the findings in the present study is the lack of a subsequent in vitro cell culture assessment to understand the potential clinical implications of the differences in the predominantly A-type and predominantly B-type AB-type carbonated apatite oxide layers formed on the different titanium alloy substrates. In future studies, we plan to assess osteoblast cell viability and early differentiation in response to the different anodized oxide groups. However, the combination of AB-type carbonated apatite-containing oxide formation, sustained oxide Ca, P, and Mg release over 7 days, and each oxide group’s early apatite-forming ability suggests that the present study’s anodized oxides may be applicable to improve outcomes across a wide variety of implant applications.

## 4. Conclusions

This study used novel citric-acid-based anodization processes to form oxides on solid and 3D-printed titanium alloy implant substrates. A combination of EDS, XPS, and XRD analyses showed that the oxides formed on each substrate incorporated substantial levels of Ca, P, and Mg bone-mineral dopants, as well as hydroxyapatite. Each oxide group was also shown to exhibit complex macro-, micro-, and nano-scaled surface topographies. XPS analyses of the outermost oxide surfaces showed lower Ca and P dopant levels for the 3D-printed oxide groups compared to those of the solid substrate oxide groups. FTIR analyses revealed predominantly B-type carbonated apatite to be incorporated into the 3D-printed diamond group oxides, while predominantly A-type carbonated apatite was incorporated into the solid group and 3D-printed gyroid group oxides. A subsequent coating dissolution assessment showed sustained Ca, P, and Mg ion release levels from each oxide group over a 7-day test period. Finally, each oxide group showed increased hydroxyapatite formation during a 7-day SBF soak, indicating good early apatite-forming ability. Overall, the findings of this study indicate that the novel anodized oxides formed on solid and 3D-printed porous titanium substrates show promise to improve the clinical outcomes of future titanium implants.

## 5. Patents

This research work is a part of patent application PCT/US25/18963.

## Figures and Tables

**Table 1:** Anodization process electrolyte composition.

Solution Component	Citric Acid (M)	Magnesium Phosphate (M)	Calcium Acetate (M)
500 mL Distilled water	0.050	0.150	0.275

## Data Availability

The data that support the findings of this study are available from the corresponding author upon reasonable request.

## References

[1] Jiang P., Zhang Y., Hu R., Shi B., Zhang L., Huang Q., Yang Y., Tang P., Lin C. (2023). Advanced surface engineering of titanium materials for biomedical applications: From static modification to dynamic responsive regulation. Bioact. Mater..

[2] Alipal J., Lee T.C., Koshy P., Abdullah H.Z., Idris M.I. (2021). Evolution of anodised titanium for implant applications. Heliyon.

[3] Alipal J., Lee T.C., Koshy P., Abdullah H.Z., Idris M.I. (2021). Influence of altered Ca-P based electrolytes on the anodised titanium bioactivity. Surf. Coat. Technol..

[4] Xiao R., Feng X., Fan R., Chen S., Song J., Gao L. (2020). 3D printing of titanium-coated gradient composite lattices for lightweight mandibular prosthesis. Compos. Part B Eng..

[5] Maietta S., Gloria A., Improta G., Richetta M., De Santis R., Martorelli M. (2019). A Further Analysis on Ti6Al4V Lattice Structures Manufactured by Selective Laser Melting. J. Healthc. Eng..

[6] Mahmoud D., Elbestawi M.A. (2017). Lattice Structures and Functionally Graded Materials Applications in Additive Manufacturing of Orthopedic Implants: A Review. J. Manuf. Mater. Process..

[7] Sing S.L., Yeong W.Y., Wiria F., Tay B. (2016). Characterization of Titanium Lattice Structures Fabricated by Selective Laser Melting Using an Adapted Compressive Test Method. Exp. Mech..

[8] Zhao P., Liu Y., Li T., Zhou Y., Leeflang S., Chen L., Wu C., Zhou J., Huan Z. (2020). 3D printed titanium scaffolds with ordered TiO2 nanotubular surface and mesoporous bioactive glass for bone repair. Prog. Nat. Sci. Mater. Int..

[9] Maconachie T., Leary M., Lozanovski B., Zhang X., Qian M., Faruque O., Brandt M. (2019). SLM lattice structures: Properties, performance, applications and challenges. Mater. Des..

[10] Riva L., Ginestra P.S., Ceretti E. (2021). Mechanical characterization and properties of laser-based powder bed–fused lattice structures: A review. Int. J. Adv. Manuf. Technol..

[11] Arabnejad S., Johnston B., Tanzer M., Pasini D. (2016). Fully porous 3D printed titanium femoral stem to reduce stress-shielding following total hip arthroplasty. J. Orthop. Res..

[12] Jetté B., Brailovski V., Dumas M., Simoneau C., Terriault P. (2018). Femoral stem incorporating a diamond cubic lattice structure: Design, manufacture and testing. J. Mech. Behav. Biomed. Mater..

[13] Kelly C., Adams S.B. (2024). 3D Printing Materials and Technologies for Orthopaedic Applications. J. Orthop. Trauma.

[14] Distefano F., Pasta S., Epasto G. (2023). Titanium Lattice Structures Produced via Additive Manufacturing for a Bone Scaffold: A Review. J. Funct. Biomater..

[15] Kelly C.N., Francovich J., Julmi S., Safranski D., Guldberg R.E., Maier H.J., Gall K. (2019). Fatigue behavior of As-built selective laser melted titanium scaffolds with sheet-based gyroid microarchitecture for bone tissue engineering. Acta Biomater..

[16] Kelly C.N., Miller A.T., Hollister S.J., Guldberg R.E., Gall K. (2018). Design and Structure–Function Characterization of 3D Printed Synthetic Porous Biomaterials for Tissue Engineering. Adv. Healthc. Mater..

[17] Yan C., Hao L., Hussein A., Young P. (2015). Ti-6Al-4V triply periodic minimal surface structures for bone implants fabricated via selective laser melting. J. Mech. Behav. Biomed. Mater..

[18] Kheradmandfard M., Fathi M.H., Ansari F., Ahmadi T. (2016). Effect of Mg content on the bioactivity and biocompatibility of Mg-substituted fluorapatite nanopowders fabricated via mechanical activation. Mater. Sci. Eng. C Mater. Biol. Appl..

[19] Jeong J., Kim J.H., Shim J.H., Hwang N.S., Heo C.Y. (2019). Bioactive calcium phosphate materials and applications in bone regeneration. Biomater. Res..

[20] Zhu Y., Su J., Qi T., Zhang G., Liu P., Qin H., Yang Q., Yao S., Zheng Y., Weng J. (2025). Research progress on the role and mechanism of magnesium-containing materials in bone repair. Biomater. Transl..

[21] Su Y., Cockerill I., Zheng Y., Tang L., Qin Y.X., Zhu D. (2019). Biofunctionalization of metallic implants by calcium phosphate coatings. Bioact. Mater..

[22] Urquia Edreira E.R., Wolke J.G.C., Aldosari A.A., Al-Johany S.S., Anil S., Jansen J.A., Beucken J.J.J.P.v.D. (2015). Effects of calcium phosphate composition in sputter coatings on in vitro and in vivo performance. J. Biomed. Mater. Res. Part A.

[23] Hu J., Shao J., Huang G., Zhang J., Pan S. (2023). In Vitro and In Vivo Applications of Magnesium-Enriched Biomaterials for Vascularized Osteogenesis in Bone Tissue Engineering: A Review of Literature. J. Funct. Biomater..

[24] Ciosek Ż., Kot K., Kosik-Bogacka D., Łanocha-Arendarczyk N., Rotter I. (2021). The Effects of Calcium, Magnesium, Phosphorus, Fluoride, and Lead on Bone Tissue. Biomolecules.

[25] Liu S.M., Li B.E., Liang C.Y., Wang H.S., Qiao Z.X. (2016). Formation mechanism and adhesive strength of a hydroxyapatite/TiO_2_ composite coating on a titanium surface prepared by micro-arc oxidation. Appl. Surf. Sci..

[26] Alipal J., Saidin S., Lo A.Z.K., Koshy P., Abdullah H., Idris M., Lee T. (2023). surface efficacy of CaP-based anodised titanium for bone implants. Surf. Interfaces.

[27] Tite T., Popa A.C., Balescu L.M., Bogdan I.M., Pasuk I., Ferreira J.M.F., Stan G.E. (2018). Cationic Substitutions in Hydroxyapatite: Current Status of the Derived Biofunctional Effects and Their In Vitro Interrogation Methods. Materials.

[28] Madupalli H., Pavan B., Tecklenburg M.M.J. (2018). Carbonate substitution in the mineral component of bone: Discriminating the structural changes, simultaneously imposed by carbonate in A and B sites of apatite. J. Solid State Chem..

[29] Alipal J., Pu’ad N.A.S.M., Nayan N.H.M., Sahari N., Abdullah H., Idris M., Lee T. (2021). An updated review on surface functionalisation of titanium and its alloys for implants applications. Mater. Today-Proc..

[30] Wang X.L., Li B.E., Zhou L.X., Ma J., Zhang X., Li H., Liang C., Liu S., Wang H. (2018). Influence of surface structures on biocompatibility of TiO_2_/HA coatings prepared by MAO. Mater. Chem. Phys..

[31] Lee T.C., Abdullah H.Z., Koshy P., Idris M.I. (2018). Deposition of novel bioactive nanoflower-like sodium titanate on TiO_2_ coating via anodic oxidation for biomedical applications. Mater. Lett..

[32] Nelson J., Jain S., Pal P., Johnson H.A., Nobles K.P., Janorkar A.V., Williamson R.S., Roach M.D. (2022). Anodized titanium with calcium and phosphorus surface enhancements for dental and orthopedic implant applications. Thin Solid Films.

[33] Jain S., Williamson R.S., Janorkar A.V., Griggs J.A., Roach M.D. (2019). Osteoblast response to nanostructured and phosphorus-enhanced titanium anodization surfaces. J. Biomater. Appl..

[34] Parekh A., Janorkar A.V., Roach M.D. (2025). Bone-like Carbonated Apatite Titanium Anodization Coatings Produced in Citrus sinensis-Based Electrolytes. Appl. Sci..

[35] Parekh A., Knotts P., Janorkar A.V., Roach M.D. (2025). Citrus Fruit-Based Calcium Titanate Anodization Coatings on Titanium Implants. Oxygen.

[36] Parekh A., Tahincioglu A., Walters C., Chisolm C., Williamson S., Janorkar A.V., Roach M.D. (2025). Citrus-Fruit-Based Hydroxyapatite Anodization Coatings on Titanium Implants. Materials.

[37] Parekh A., Moore M., Janorkar A.V., Roach M.D. (2024). Mg-Doped Carbonated Hydroxyapatite and Tricalcium Phosphate Anodized Coatings on Titanium Implant Alloys. Appl. Sci..

[38] Ettuthaiyil Sambasivan A., Parekh A., Janorkar A.V., Roach M.D. (2025). Organic Acid-Based Anodization Process to Produce Bioactive Oxides on Titanium Implants. Materials.

[39] (2014). Implants for Surgery—In Vitro Evaluation for Apatite-Forming Ability of Implant Materials.

[40] Stępniowski W.J., Michalska-Domańska M., Norek M., Twardosz E., Florkiewicz W., Polkowski W., Zasada D., Bojar Z. (2014). Anodization of cold deformed technical purity aluminum (AA1050) in oxalic acid. Surf. Coat. Technol..

[41] Navidirad M., Stepniowski W.J., Cartier E., Christ T., Watanabe M., Misiolek W.Z. (2021). Investigation on the Strain Induced Oxide Layer Fracture and Bonding During Cold Rolling of Aluminum Alloys.

[42] Cruz M.B., Silva N., Marques J.F., Mata A., Silva F.S., Caramês J. (2022). Biomimetic Implant Surfaces and Their Role in Biological Integration-A Concise Review. Biomimetics.

[43] Lin D.J., Fuh L.J., Chen W.C. (2020). Nano-morphology, crystallinity and surface potential of anatase on micro-arc oxidized titanium affect its protein adsorption, cell proliferation and cell differentiation. Mater. Sci. Eng. C Mater. Biol. Appl..

[44] Wu S.L., Weng Z.Y., Liu X.M., Yeung K.W.K., Chu P.K. (2014). Functionalized TiO_2_ Based Nanomaterials for Biomedical Applications. Adv. Funct. Mater..

[45] Gomes G.C., Borges F.O., Borghi F.F., Cavalcanti G., Martins C., Palleschi V., Mello A. (2021). Rapid stoichiometric analysis of calcium-phosphorus ratio on hydroxyapatite targets by one-point calibration laser-induced breakdown spectroscopy. Spectrochim. Acta Part B At. Spectrosc..

[46] Zhu X., Chen J., Scheideler L., Reichl R., Geis-Gerstorfer J. (2004). Effects of topography and composition of titanium surface oxides on osteoblast responses. Biomaterials.

[47] Sul Y.T., Johansson C.B., Petronis S., Krozer A., Jeong Y., Wennerberg A., Albrektsson T. (2002). Characteristics of the surface oxides on turned and electrochemically oxidized pure titanium implants up to dielectric breakdown: The oxide thickness, micropore configurations, surface roughness, crystal structure and chemical composition. Biomaterials.

[48] Arce J.E., Arce A.E., Aguilar Y., Yate L., Moya S., Rincón C., Gutiérrez O. (2016). Calcium phosphate-calcium titanate composite coatings for orthopedic applications. Ceram. Int..

[49] Frauchiger V.M., Schlottig F., Gasser B., Textor M. (2004). Anodic plasma-chemical treatment of CP titanium surfaces for biomedical applications. Biomaterials.

[50] Durdu S., Deniz Ö.F., Kutbay I., Usta M. (2013). Characterization and formation of hydroxyapatite on Ti6Al4V coated by plasma electrolytic oxidation. J. Alloys Compd..

[51] Ohtsu N., Ito A., Saito K., Hanawa T. (2007). Characterization of calcium titanate thin films deposited on titanium with reactive sputtering and pulsed laser depositions. Surf. Coat. Technol..

[52] Sul Y.T. (2003). The significance of the surface properties of oxidized titanium to the bone response: Special emphasis on potential biochemical bonding of oxidized titanium implant. Biomaterials.

[53] Shen Z., Xu Y., Qian X.N., Zhou Y., Zhou Y., Zhou J., Liu Y., Zhang S., Qiu J. (2024). Enhanced osteogenic and antibacterial properties of titanium implant surface modified with Zn-incorporated nanowires: Preclinical in vitro and in vivo investigations. Clin. Oral Implant. Res..

[54] Popa C., Ciobanu C., Iconaru S.L., Stan M., Dinischiotu A., Negrila C., Motelica-Heino M., Guegan R., Predoi D. (2014). Systematic investigation and in vitro biocompatibility studies on mesoporous europium doped hydroxyapatite. Cent. Eur. J. Chem..

[55] Lin C., Zhang H., Zhang J., Chen C. (2019). Enhancement of the Humidity Sensing Performance in Mg-Doped Hexagonal ZnO Microspheres at Room Temperature. Sensors.

[56] Cimpeanu C., Predoi D., Ciobanu C.S., Iconaru S.L., Rokosz K., Predoi M.V., Raaen S., Badea M.L. (2024). Development of Novel Biocomposites with Antimicrobial-Activity-Based Magnesium-Doped Hydroxyapatite with Amoxicillin. Antibiotics.

[57] Bhatnagar D., Gautam S., Sonowal L., Bhinder S.S., Ghosh S., Pati F. (2024). Enhancing Bone Implants: Magnesium-Doped Hydroxyapatite for Stronger, Bioactive, and Biocompatible Applications. ACS Appl. Bio Mater..

[58] Berzina-Cimdina L., Borodajenko N., Theophile T. (2012). Research of Calcium Phosphates Using Fourier Transform Infrared Spectroscopy. Infrared Spectroscopy—Materials Science, Engineering and Technology.

[59] Liu S.M., Yang X.J., Cui Z.D., Zhu S.L., Wei Q.A. (2011). One-step synthesis of petal-like apatite/titania composite coating on a titanium by micro-arc oxidation. Mater. Lett..

[60] Wang S., Zhao X., Hsu Y., He Y., Wang F., Yang F., Yan F., Xia D., Liu Y. (2023). Surface modification of titanium implants with Mg-containing coatings to promote osseointegration. Acta Biomater..

[61] Park J.W., Hanawa T., Chung J.H. (2019). The relative effects of Ca and Mg ions on MSC osteogenesis in the surface modification of microrough Ti implants. Int. J. Nanomed..

[62] Sheng X., Wang A., Wang Z., Liu H., Wang J., Li C. (2022). Advanced Surface Modification for 3D-Printed Titanium Alloy Implant Interface Functionalization. Review. Front. Bioeng. Biotechnol..

[63] Qin Z., He Y., Gao J., Dong Z., Long S., Cheng L., Shi Z. (2023). Surface modification improving the biological activity and osteogenic ability of 3D printing porous dental implants. Front. Mater..

